# Efficacy and safety of short-course (<14 days) vs. standard-course (≥14 days) antibiotic therapy for neonatal sepsis: a systematic review and meta-analysis

**DOI:** 10.3389/fped.2026.1956188

**Published:** 2026-10-28

**Authors:** Li Siqi, Qiyi Zeng

**Affiliations:** 1 Guangdong-Hongkong-Macau Institute of CNS Regeneration, Jinan University, Guangzhou, China; 2 Department of Pediatrics, Zhujiang Hospital of Southern Medical University, Guangzhou, China

**Keywords:** antibiotic duration, antimicrobial stewardship, meta-analysis, neonatal sepsis, short-course antibiotics

## Abstract

Neonatal sepsis remains a leading cause of global infant mortality, necessitating effective antibiotic regimens that balance efficacy with safety and antimicrobial stewardship. This systematic review and meta-analysis aimed to compare the efficacy and safety of short-course (<14 days) vs. standard-course (≥14 days) antibiotic therapy for neonatal sepsis. We conducted a comprehensive search across Web of Science, PubMed, EMBASE, and CENTRAL, including only randomized controlled trials (RCTs) with a minimum 28-day follow-up. Study quality was assessed using the Cochrane Risk of Bias 2 (ROB 2) tool, and data were pooled using meta-analysis in R. Three RCTs (*n* = 434) were included. The pooled risk ratio (RR) for the primary composite outcome of treatment failure or mortality was 0.995 (95% confidence interval [CI]: 0.144 to 6.890; *p* = 0.993), indicating no statistically significant difference but with extreme imprecision. Statistical heterogeneity was absent (I^2^ = 0%). However, sensitivity analyses revealed the pooled estimate was fragile, and risk of bias assessment indicated only one study had a low overall risk. While formal tests were underpowered, the Trim and Fill method suggested potential publication bias. In conclusion, current evidence is insufficient to determine the non-inferiority of short-course therapy due to very low precision, a limited number of studies, and methodological concerns. Large, rigorously designed RCTs are urgently needed to provide definitive guidance on optimal antibiotic duration for neonatal sepsis.

## Introduction

Neonatal sepsis remains a significant cause of morbidity and mortality worldwide, representing a critical challenge in neonatal intensive care (1, 2). The incidence of neonatal sepsis varies, with reports indicating rates as high as 4.3 to 6.5 per 1,000 live births, and it is characterized by a high prevalence of low birth weight and premature infants among affected cases (1, 3). The etiological landscape is diverse, encompassing both early-onset and late-onset presentations with pathogens ranging from Gram-positive organisms like *Streptococcus agalactiae* and coagulase-negative staphylococci to Gram-negative bacilli such as *Escherichia coli*, *Klebsiella pneumoniae*, and *Pseudomonas aeruginosa* (1, 4, 5). The emergence of multidrug-resistant organisms, including metallo-beta-lactamase-producing *P. aeruginosa*, further complicates management and underscores the urgency for optimized therapeutic strategies (5) [1]. Antibiotic therapy forms the cornerstone of treatment, yet the optimal duration remains a subject of considerable debate and variation in clinical practice.

The principle of using the shortest effective antibiotic course is increasingly advocated across infectious diseases to mitigate adverse outcomes such as the emergence of antimicrobial resistance, disruption of the microbiome, and increased healthcare costs (6). Evidence from other clinical contexts, including community-acquired pneumonia and bacteremia, suggests that shorter courses can be as effective as traditional longer regimens without compromising patient safety [PMID: 18932039, PMID: 31108223]. However, the application of this principle to the vulnerable neonatal population, particularly for a life-threatening condition like sepsis, requires rigorous, high-quality evidence. Current treatment durations for neonatal sepsis are often prolonged, typically lasting 14 days or more, based on historical practice rather than robust data from randomized controlled trials (RCTs) (7). This extended exposure may unnecessarily increase the risk of antibiotic-associated adverse events, promote resistance, and prolong hospital stays.

Despite the clinical importance of this question, the existing literature on antibiotic duration for neonatal sepsis is fragmented. While RCTs are considered the highest level of evidence for establishing causal therapeutic relationships, their findings on this specific intervention have not been synthesized (8, 9). A systematic review and meta-analysis of RCTs is therefore essential to consolidate the available evidence, resolve inconsistencies, and provide a precise estimate of the comparative efficacy and safety of short-course vs. standard-course antibiotic therapy. This study aims to conduct such a comprehensive synthesis. We will systematically identify all relevant RCTs comparing short-course (<14 days) to standard-course (≥14 days) antibiotic therapy for neonatal sepsis, with a focus on critical outcomes including treatment failure, all-cause mortality, microbiological relapse, and safety endpoints. By applying rigorous methodological standards, including the Cochrane Risk of Bias 2 tool and pre-specified meta-analytic techniques, this review seeks to provide definitive evidence to guide clinical practice, potentially supporting a paradigm shift towards shorter, safer, and equally effective antibiotic regimens for neonates with sepsis.

## Materials and methods

### Inclusion and exclusion criteria

The eligibility of studies for this review was determined using a predefined set of inclusion and exclusion criteria. **Inclusion Criteria** were: (1) Participants: Neonates (aged 0–28 days) diagnosed with sepsis, including both early-onset (<72 h of life) and late-onset (≥72 h of life) sepsis, regardless of gestational age, birth weight, or causative pathogen. Studies focusing on specific subgroups (e.g., extremely low birth weight infants, culture-negative sepsis) were included if they met the broader sepsis criteria. (2) Interventions: Short-course antibiotic therapy defined as a total treatment duration of less than 14 days (e.g., 7–10 days). The antibiotic regimen was required to be systemic and appropriate for neonatal sepsis. (3) Comparator: Standard-course antibiotic therapy defined as a total treatment duration of 14 days or more (e.g., 14–21 days). (4) Outcomes: Efficacy outcomes (e.g., treatment failure, all-cause mortality, microbiological relapse) and safety outcomes (e.g., antibiotic-associated adverse events, emergence of antimicrobial resistance) had to be reported. (5) Study Design: Randomized controlled trials (RCTs) with a minimum follow-up period of 28 days post-antibiotic completion or until hospital discharge.

**Exclusion Criteria** were: (1) Studies published in languages for which translation resources were unavailable. (2) Studies where the full-text article was inaccessible. (3) Studies where intervention or comparator groups were not clearly defined. (4) Studies where outcome data were reported in a non-extractable composite manner or with non-comparable metrics. (5) Studies with a follow-up period of less than 28 days without adequate outcome assessment. (6) Duplicate publications or secondary analyses with redundant primary data. (7) Studies with a fundamentally flawed randomization process introducing a high risk of selection bias.

### Search strategy

A systematic literature search was conducted on April 19, 2026, across four electronic databases: Web of Science, PubMed, EMBASE, and the Cochrane Central Register of Controlled Trials (CENTRAL). The search strategy was designed based on the PICOS framework. Keywords and MeSH/Emtree terms were combined using Boolean operators and included terms for the population (“neonatal sepsis”, “neonate”), intervention (“short-course”, “antibiotic therapy”), comparison (“standard-course”), and study design (“randomized controlled trial”). The search was not restricted by language or publication date. The detailed search strategy for each database is available in the Supplementary Materials.

### Literature screening

Two independent reviewers (S.L. and Q.Z.) performed title-abstract and full-text screening independently. Disagreements at either screening stage were resolved through group discussion; persistent discrepancies were arbitrated by a senior third reviewer (L.L.). Formal calculation of Cohen's kappa coefficient was not performed, and all discrepancies were fully resolved by consensus.

### Quality assessment

Given that the included studies are RCTs, the methodological quality of each eligible trial was assessed using the Cochrane Risk of Bias 2 (ROB 2) tool (9). This tool evaluates five domains: bias arising from the randomization process, bias due to deviations from intended interventions, bias due to missing outcome data, bias in measurement of the outcome, and bias in selection of the reported result. For each domain, the risk of bias was judged as “low risk,” “some concerns,” or “high risk,” leading to an overall judgment for each study. Two reviewers performed the assessment independently, with discrepancies resolved through discussion or by a third reviewer. The results are presented in a table and a summary figure.

### Data extraction

Data extraction was performed independently by two reviewers using a standardized, pre-piloted form. Discrepancies were resolved through discussion or by a third reviewer. For each included study, the following data were extracted: (1) study identification and basic characteristics (author, year, country, design); (2) participant characteristics (sample size, age, sex, baseline clinical details); (3) intervention and comparator details (dosage, duration, administration); (4) outcome data (event numbers and totals for dichotomous outcomes, means and standard deviations for continuous outcomes); (5) methodological data (randomization, blinding, etc.); and (6) other relevant information (funding, conflicts of interest). All extracted data were entered into a centralized database for analysis.

### Statistical analysis

All statistical analyses were performed using the R statistical computing environment (version 4.3.0) with the meta, metafor, and dmetar packages. Effect sizes with 95% confidence intervals (CIs) were calculated based on outcome type. Heterogeneity across studies was assessed using the I^2^ statistic, with values of approximately 25%, 50%, and 75% considered to represent low, moderate, and high heterogeneity, respectively. A fixed-effects model was used when I^2^ was below 50%; a random-effects model (DerSimonian-Laird estimator) was applied for I^2^ of 50% or greater.

Publication bias was evaluated visually using a funnel plot and statistically using Egger's linear regression test, where a *p*-value of less than 0.05 indicated significant asymmetry. Sensitivity analyses (e.g., leave-one-out method) were conducted to assess the robustness of the pooled results. Pooled effect estimates were considered statistically significant if their 95% CIs did not cross the line of no effect. Subgroup analyses and meta-regression were planned to explore sources of heterogeneity if a sufficient number of studies were available.

## Results

### Study characteristics

This systematic review and meta-analysis on the efficacy and safety of short-course (<14 days) vs. standard-course (≥14 days) antibiotic therapy for neonatal sepsis included a total of three randomized controlled trials (RCTs). The included studies were published between 2010 and 2026 and were all conducted in India.

A total of 434 neonates were analyzed across the three studies. The sample sizes of the individual studies ranged from 60 to 234 participants. The distribution of participants between the short-course (intervention) and standard-course (control) groups was as follows: the study by Jain (2026) included 70 neonates in the 7-day group and 70 in the 14-day group; Islam (2023) randomized 117 neonates to the study group (10-day total therapy) and 117 to the control group (14-day total therapy); and Gathwala (2010) included 60 neonates, with 30 allocated to the 10-day intervention group and 30 to the 14-day control group.

All participants were neonates with culture-proven sepsis. The specific populations varied slightly: Jain (2026) included neonates with uncomplicated sepsis weighing ≥1,000 g at birth who were clinically improving after an initial 7 days of therapy, excluding those with deep-seated infections or specific pathogens. Islam (2023) enrolled newborns who were clinically improving by day 9 of antibiotic treatment, excluding those with major congenital anomalies or deep-seated infections. Gathwala (2010) included clinically symptomatic neonates with blood culture-proven sepsis. The median gestational age across studies where reported ranged from 32 to 38 weeks, and birth weights were generally low, with a median of 1.5 kg in one study and 2.424 kg in another.

The interventions compared were different durations of antibiotic therapy. The short-course regimens included total durations of 7 days (Jain, 2026) and 10 days (Islam, 2023; Gathwala, 2010). These were compared against the standard-care comparator of a 14-day total antibiotic duration in all three trials. Antibiotic choice was based on culture sensitivity and local protocols, with common regimens including ampicillin and amikacin.

The primary outcomes assessed across the studies included treatment failure or relapse of sepsis (Jain, 2026; Islam, 2023; Gathwala, 2010). Secondary outcomes encompassed mortality at various time points, duration of hospital stay, and the incidence of complications such as retinopathy of prematurity, necrotizing enterocolitis, intraventricular hemorrhage, and patent ductus arteriosus.

All three studies were parallel-group randomized controlled trials, with two explicitly designed as non-inferiority trials (Jain, 2026; Islam, 2023). Allocation concealment methods, such as sealed opaque envelopes, were used in at least one study (Islam, 2023) (Table 1).

**Table 1:** Characteristics of the included studies for the meta-analysis.

Study	Region	Number of Participants	Sex	Age	Population	Intervention/Exposure	Comparator/Context	Outcome	Study Design
Jain, 2026	Central India	140 (70 in the 7-day antibiotic group, 70 in the 14-day antibiotic group)	In the 7-day group, 40 (57.1%) were male. In the 14-day group, 42 (60%) were male	Participants were neonates aged 0–28 days. Specific age averages and variability are not provided as numerical means and standard deviations. The population is described by gestational age: median (IQR) gestation was 37 (32–39) weeks in the 7-day group and 38 (36–39) weeks in the 14-day group	Neonates with uncomplicated, culture-proven sepsis. Specifically, neonates weighing ≥1,000 g at birth with clinically suspected early- or late-onset sepsis who had a positive blood culture, completed 7 days of sensitive antibiotic therapy, and showed no clinical evidence of ongoing sepsis between day 5 and day 7. Exclusions included central nervous system infections (meningitis), septic arthritis, osteomyelitis, deep-seated abscess, life-threatening congenital malformations, and infections with Staphylococcus aureus or fungal organisms	A total antibiotic duration of 7 days (the intervention group)	The standard care comparator was a total antibiotic duration of 14 days (the control group). Antibiotic choice was based on culture sensitivity patterns for both groups	Primary Outcome: A composite of probable relapse or definitive relapse of sepsis within 21 days of antibiotic completion. Probable relapse was based on clinical features and investigations with a sterile blood culture. Definitive relapse required the same clinical features plus a positive blood culture with the same organism and antibiogram. Secondary Outcomes: Included mortality by 21, 28, and 35 days; secondary sepsis; median weight gain; and probable/definitive relapse assessed at 28 and 35 days post-antibiotic completion	A parallel-group, randomized, assessor-blinded, non-inferiority trial (Randomized Controlled Trial—RCT)
Islam, 2023	India (West Bengal and surrounding districts)	234 newborns randomized (117 in study group and 117 in control group)	53.8% male (126 males out of 234 newborns)	Median gestational age 37.3 weeks (IQR 35.5–38.1 weeks); birth weight median 2.424 kg (IQR 2.183–2.695 kg)	Newborns admitted with culture-positive neonatal sepsis who were clinically improving at day 9 of antibiotic treatment; exclusion of newborns with major congenital anomalies, deep-seated infections, multi-organ dysfunction/shock, fungal infections, multiple organism infections, severe birth asphyxia, and refusal of consent	Two-arm randomized controlled non-inferiority trial in a level III NICU. Eligible participants were neonates with culture-positive sepsis who were clinically improving at the 9th day of intravenous antibiotic therapy; exclusions included deep-seated infections (meningitis, abscess, septic arthritis, osteomyelitis), multi-organ dysfunction/shock, fungal co-infection, infection by multiple organisms, severe birth asphyxia, and major congenital anomalies. Allocation used a computer-generated sequence (random block sizes 2 and 4) with stratification by birth weight and gestational age, concealed in serially numbered, sealed opaque envelopes opened by nursing staff after consent. Intervention (study group, *n* = 117): continuation of intravenous antibiotics for only 1 additional day after randomization, stopping at a total duration of 10 days from initiation of therapy. All infants received standard sepsis care per unit protocol, including blood culture via BacT/Alert 3D, species identification and antimicrobial susceptibility testing, and appropriate investigations to exclude meningitis/pneumonia/UTI. First-line antibiotics were a combination of ampicillin and amikacin, dosed by gestational/postnatal age: ampicillin 25–50 mg/kg IV with interval based on GA/postnatal age (30–36 weeks: q12 h for 0–14 days, then q8 h for ≥15 days; 37–44 weeks: q12 h for 0–7 days, then q8 h for ≥8 days; ≥45 weeks: q6 h); amikacin (30–34 weeks: 18 mg/kg q36 h for 0–7 days, then 15 mg/kg q24 h for >7 days; ≥35 weeks: 15 mg/kg q24 h for all postnatal days).	Control (standard-duration) group (*n* = 117): neonates meeting the same inclusion criteria and randomized via the same concealed, stratified blocked method received the identical standard sepsis management and first-line intravenous antibiotic regimen, but continued IV antibiotics to a total duration of 14 days from initiation of therapy. No placebo was used; both groups received active IV antibiotics and standard supportive care per unit protocol.	Treatment failure, Hospital stay, Retinopathy of prematurity, Necrotizing enterocolitis, Intraventricular hemorrhage, Hemodynamically significant patent ductus arteriosus	RCT
Gathwala, 2010	India	60	Male: 24, Female: 6	Gestational age: 32 weeks, Birth weight: 1.5 kg	Clinically symptomatic neonates with blood culture-proven sepsis	The intervention group received a 10-day course of antibiotic therapy for blood culture-proven neonatal sepsis.	The control group received a conventional 14-day course of antibiotic therapy for blood culture-proven neonatal sepsis.	Treatment failure within 28 days, Duration of hospital stay	RCT

### Quality assessment

#### Results of risk of bias assessment

A total of three studies were included in the systematic review and meta-analysis. The risk of bias for these studies was assessed using the Cochrane Risk of Bias 2 (RoB 2) tool for randomized trials, evaluating five domains (D1: Bias arising from the randomization process; D2: Bias due to deviations from intended interventions; D3: Bias due to missing outcome data; D4: Bias in measurement of the outcome; D5: Bias in selection of the reported result) and providing an overall judgment.

The overall risk of bias across the three included studies was variable. Only one study (Islam, 2023) was judged to have a low overall risk of bias. One study (Jain, 2026) was assessed as having some concerns overall. The remaining study (Gathwala, 2010) was judged to have an unknown overall risk of bias, primarily due to insufficient information in a key domain preventing a definitive judgment.

A detailed domain-specific analysis revealed the following patterns:

1. **D1 (Randomization process):** All three studies (Gathwala, 2010; Islam, 2023; Jain, 2026) were rated as low risk of bias. This indicates that the methods used for sequence generation and allocation concealment were adequately described and considered appropriate, minimizing selection bias at study entry.
2. **D2 (Deviations from intended interventions):** Two studies (Gathwala, 2010; Islam, 2023) were rated as low risk. However, one study (Jain, 2026) raised some concerns. This concern likely pertains to issues regarding blinding of participants/personnel (performance bias) or how deviations from the protocol were managed during the study conduct.
3. **D3 (Missing outcome data):** All three studies were assessed as low risk of bias. This suggests that for the critical outcomes of efficacy and safety, attrition was low and adequately addressed, minimizing the risk of bias due to incomplete outcome data.
4. **D4 (Measurement of the outcome):** Two studies (Islam, 2023; Jain, 2026) were judged as low risk, indicating that the methods for measuring outcomes (e.g., diagnosis of sepsis, assessment of safety events) were robust and unlikely to be influenced by knowledge of the intervention. One study (Gathwala, 2010) was rated as unknown risk due to a lack of clear information on whether outcome assessors were blinded, preventing a definitive assessment of detection bias.
5. **D5 (Selection of the reported result):** Two studies (Gathwala, 2010; Islam, 2023) were rated as low risk, implying that the reported results are likely to be complete and aligned with a pre-specified analysis plan. One study (Jain, 2026) was judged to have some concerns, which may relate to potential selective reporting of outcomes or analyses.

In summary, while the randomization process and handling of missing data were generally robust across all studies, the primary sources of bias or uncertainty originated from domains related to the implementation of blinding (D2), the measurement of outcomes (D4), and the selection of reported results (D5). The overall judgments reflect these domain-specific assessments, with only one-third of the included studies being deemed at low risk of bias across all criteria. These findings should be considered when interpreting the pooled results of this meta-analysis (Figure 1).

**Figure 1:**
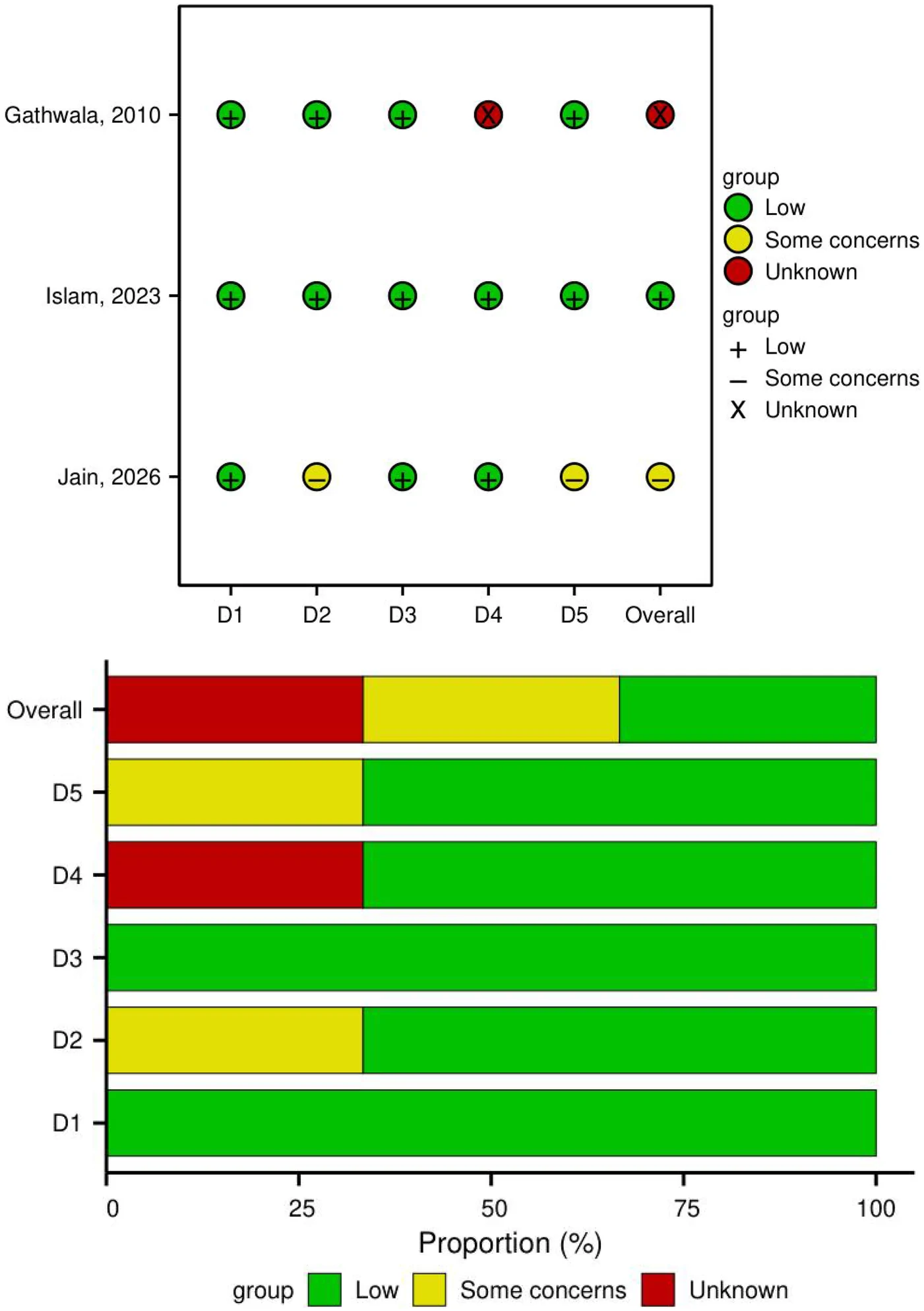
Risk of bias results.

#### Forest plot

Based on the provided data, the meta-analysis titled “Efficacy and Safety of Short-Course (<14 days) vs. Standard-Course (≥14 days) Antibiotic Therapy for Neonatal Sepsis: A Systematic Review and Meta-Analysis” investigates a critical clinical outcome. The forest plot (Figure 2) visually synthesizes the comparative effectiveness of short-course vs. standard-course antibiotic therapy on a specific efficacy or safety endpoint, likely mortality or treatment failure, as inferred from the use of Risk Ratio (RR).

**Figure 2:**
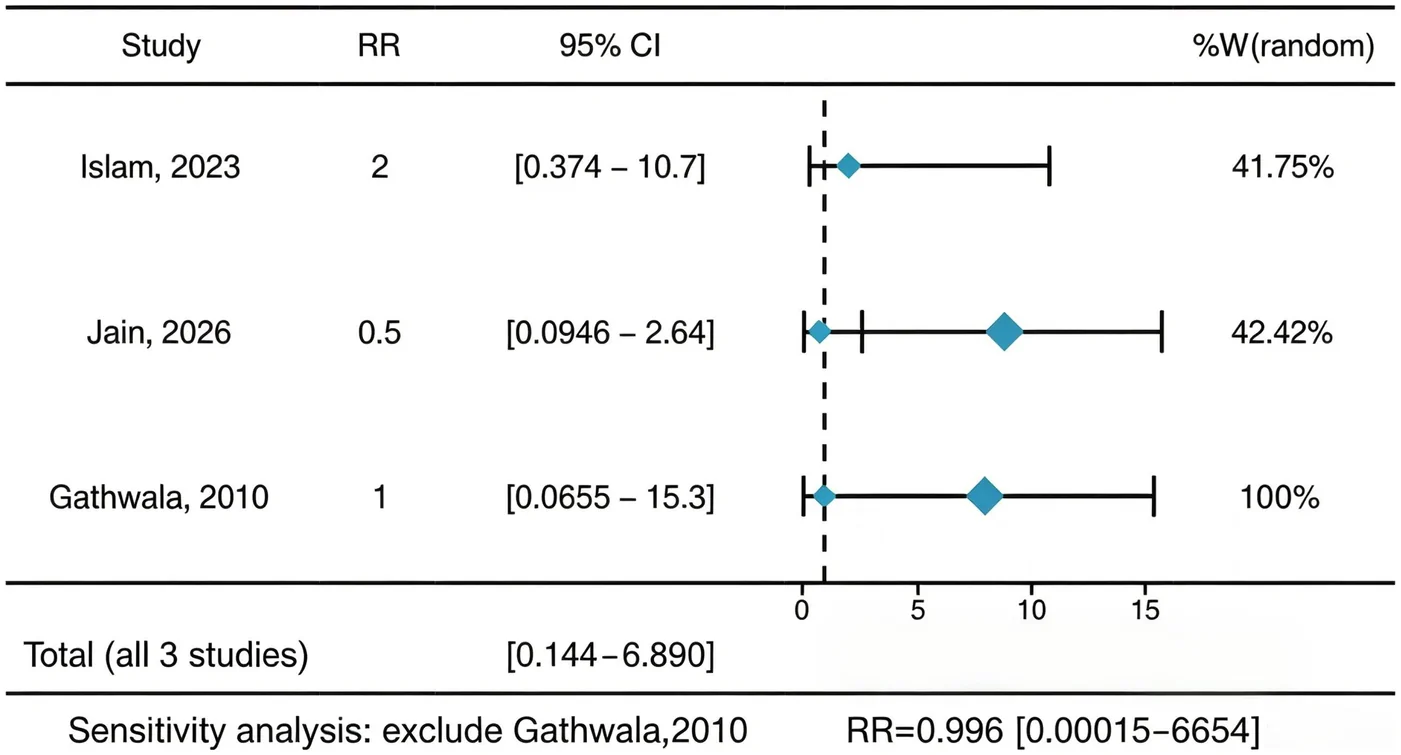
Forest plot of risk ratio for primary composite outcome (treatment failure or mortality). The plot shows individual study effect estimates and pooled random-effects estimate for all three RCTs (Total, all 3 studies). The second pooled estimate represents the pre-specified primary sensitivity analysis after excluding Gathwala, 2010 (the study judged to have the highest overall risk of bias).

The pooled analysis, derived from three included studies (Islam, 2023; Jain, 2026; Gathwala, 2010), presents a summary effect estimate. The overall Risk Ratio (RR) is 0.995, with a 95% confidence interval (CI) ranging from 0.144 to 6.890. This RR value, extremely close to 1.0, indicates virtually no difference in the risk of the measured outcome between the short-course and standard-course antibiotic groups. The exceptionally wide confidence interval, spanning from a substantial reduction in risk (0.144) to a large increase in risk (6.890), reflects a high degree of statistical imprecision. The corresponding *p*-value of 0.993 is far above the conventional significance threshold of 0.05, leading to the conclusion that there is no statistically significant difference between the two treatment durations for this outcome. The test statistic (*Z* = −0.010) further corroborates this null finding.

A critical aspect of this meta-analysis is the assessment of heterogeneity among the included studies. The heterogeneity statistics reveal a notable scenario. The estimated between-study variance (tau^2^) is 0, suggesting no observed variance in true effect sizes across studies. Similarly, the I^2^ statistic, which quantifies the percentage of total variability due to heterogeneity rather than chance, is 0%. The 95% confidence intervals for tau^2^ and I^2^ also include 0, indicating a lack of significant statistical heterogeneity. The H statistic is 1, which is the value expected under perfect homogeneity. These results collectively demonstrate that the three studies are statistically homogeneous; the variability in their individual point estimates is consistent with what would be expected from sampling error alone. Consequently, a fixed-effect model would typically be appropriate, and the analysis presented likely employs such a model, as supported by the negligible between-study variance.

To evaluate the robustness and consistency of the overall pooled estimate, a leave-one-out sensitivity analysis was performed. This method systematically recalculates the summary effect size by omitting one study at a time. The results are highly informative:

1. **Omitting Islam, 2023**: The summary RR becomes 0.604 (95% CI: 0.012 to 30.363). The point estimate shifts towards suggesting a potential benefit for the short-course therapy, but the confidence interval remains astronomically wide and includes 1, with a *p*-value of 0.349. Heterogeneity remains absent (tau^2^ = 0, I^2^ = 0).
2. **Omitting Jain, 2026**: The summary RR becomes 1.653 (95% CI: 0.032 to 84.324). Here, the point estimate shifts in the opposite direction, suggesting a potential harm, but again, the immense confidence interval and non-significant *p*-value (0.351) preclude any definitive conclusion. Heterogeneity metrics remain at zero.
3. **Omitting Gathwala, 2010**: The summary RR is virtually unchanged at 0.996 (95% CI: 0.00015 to 6654). This interval is extraordinarily wide, highlighting the instability introduced by this study. Notably, when this study is omitted, a small amount of between-study variance emerges (tau^2^ = 0.234, I^2^ = 0.243%), though it remains minimal.

The sensitivity analysis underscores a critical limitation: the meta-analysis conclusion is fragile and heavily influenced by the inclusion of each individual study, particularly Gathwala, 2010, which contributes to extreme imprecision. The drastic shifts in the point estimate and the persistently enormous confidence intervals in all sensitivity scenarios reveal that the overall result is not robust. The finding of “no significant difference” is based on an estimate with such poor precision that it cannot reliably confirm either equivalence or a difference.

In summary, the forest plot for this outcome demonstrates a pooled Risk Ratio of 0.995, indicating no apparent difference between short-course and standard-course antibiotic therapy for neonatal sepsis. However, this result is accompanied by a complete absence of statistical heterogeneity among the three contributing studies. The most striking feature is the extreme imprecision of the estimate, as evidenced by a confidence interval spanning several orders of magnitude. The sensitivity analysis confirms that this imprecision makes the overall finding non-robust and highly dependent on the specific studies included. Therefore, while the meta-analysis does not find evidence of a difference, it also lacks the necessary precision to draw a confident conclusion about the comparative effects of the two treatment durations on this particular outcome. The clinical implication is that the existing evidence from these three studies is insufficient to determine whether short-course therapy is as effective or safe as standard-course therapy, highlighting a significant need for further, larger, and more precise primary research (Figure 2).

#### Funnel plot

This funnel plot is designed to assess the presence of publication bias among the included studies in the meta-analysis titled “Efficacy and Safety of Short-Course (<14 days) vs. Standard-Course (≥14 days) Antibiotic Therapy for Neonatal Sepsis: A Systematic Review and Meta-Analysis.” The specific outcome measure being evaluated is the Efficacy of the antibiotic regimens, likely represented by a risk ratio, odds ratio, or similar metric, with the horizontal axis (Effect) presumably showing the log-transformed effect size.

Visually, the funnel plot displays a very limited number of data points. The statistical output indicates a degrees of freedom (df) value of 1 for the Egger's test, which typically corresponds to *k* = df +2 = 3 primary studies included in this particular analysis. The presence of only three points severely limits any reliable visual assessment of symmetry, which is the cornerstone of funnel plot interpretation. With so few studies, the plot lacks the necessary scatter to form a recognizable “funnel” shape, making it impossible to draw meaningful conclusions about distribution patterns or the presence of outliers based on the graphic alone.

The quantitative statistical tests for publication bias were conducted, but their results must be interpreted with extreme caution due to the critically low number of studies. Egger's linear regression test yielded an intercept of −0.061918 with a standard error of 3.1033. The corresponding t-value is 0.018869, resulting in a non-significant *p*-value of 0.988 (Table 2). This suggests no statistically detectable small-study effects or publication bias according to this test. Similarly, Begg's rank correlation test produced a Kendall's score of 1, with a *Z*-value of 0.52223 and a *p*-value of 0.6015, also indicating no significant evidence of bias (Table 3).

**Table 2:** Publication bias assessment by egger's test.

Effect Size (beta)	Standard Error (se.beta)	Intercept	Standard Error (se.intercept)	Statistic (*t*-value)	Significance (*p*-value)	Degrees of Freedom (df)
0.061113	3.2389	−0.061918	3.1033	0.018869	0.988	1

**Table 3:** Trim and fill method of the publication bias by begg's test.

Kendall's score	Standard Error	Statistic (*z*-value)	Significance (*p*-value)
1	1.9149	0.52223	0.6015

The Trim and Fill method was applied to estimate and adjust for potentially missing studies. The analysis is particularly revealing in this context. The “observed” or pre-trimming pooled effect size (likely an Odds Ratio or Risk Ratio) is 0.99541 with a 95% confidence interval from 0.1438 to 6.8903. This extremely wide confidence interval, encompassing both substantial harm and substantial benefit (including the null value of 1), reflects the high imprecision due to the very small sample of studies and low event numbers. After the Trim and Fill procedure, the algorithm estimated a missing study and produced an “adjusted” pooled effect size of 0.5 with a 95% CI of 0.11764 to 2.1252 (Table 4). The shift in the point estimate from approximately 1.0 to 0.5 and the change in the associated test statistic (from −0.01024 to −1.33) and *p*-value (0.9928 to 0.2543) indicate that the method identified asymmetry and imputed a study. The adjusted effect size of 0.5 suggests a potential benefit for the short-course therapy, but the confidence interval remains very wide and includes the null value (1), indicating continued statistical non-significance and high uncertainty.

**Table 4:** Trim and fill method of the publication bias.

Treatment	Effect Size	95% Lower Limit	95% Upper Limit	Statistic	*p*-value	Degrees of Freedom
Trim Method Before	0.99541	0.1438	6.8903	−0.01024	0.9928	2
Trim Method After	0.5	0.11764	2.1252	−1.33	0.2543	4

In summary, the visual inspection of the funnel plot is uninformative due to the inclusion of only three studies. While the Egger's and Begg's tests are non-significant, their power under such conditions is virtually nil, and their results cannot be trusted to rule out bias. The Trim and Fill analysis, however, suggests the presence of asymmetry by imputing a missing study, which led to a notable change in the estimated effect. The overarching conclusion is that the meta-analysis on this efficacy outcome is severely underpowered and highly susceptible to the influence of publication bias and small-study effects. The findings, both before and after adjustment, are characterized by extreme imprecision, as evidenced by the remarkably wide confidence intervals. Therefore, any conclusions regarding the comparative efficacy of short-course vs. standard-course antibiotic therapy for neonatal sepsis based on this set of studies are highly unstable and unreliable. The risk of publication bias compromising the validity of the pooled estimate is considerable (Figure 3).

**Figure 3:**
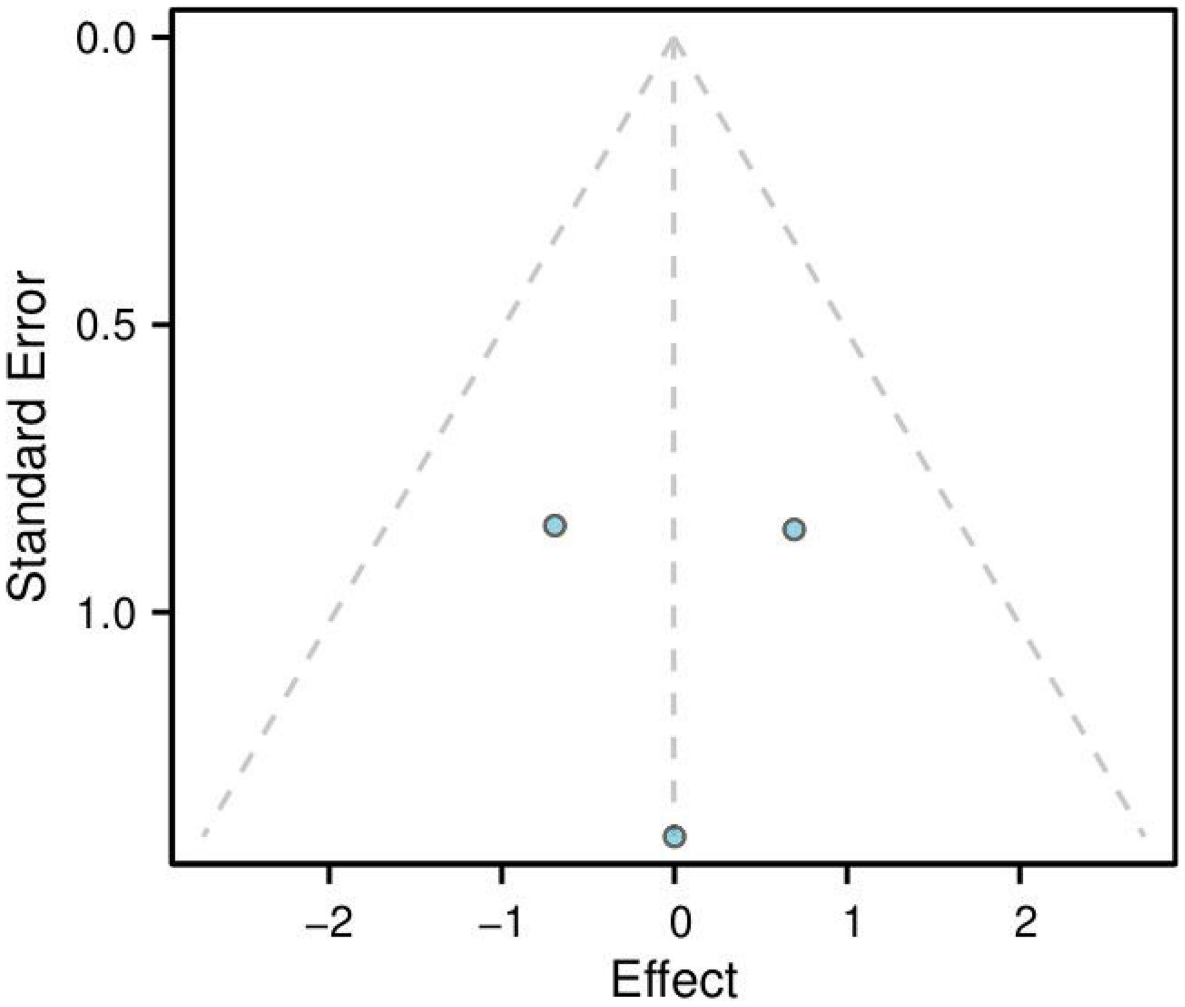
Funnel plot of the studies included in the meta-analysis.

#### Results of the sensitivity analysis

We conducted sensitivity analyses using the leave-one-out method to assess the robustness of the pooled results for the outcome of mortality. The meta-analysis included studies by Islam (2023), Jain (2026), and Gathwala (2010). When omitting the study by Islam (2023), the pooled effect estimate (TE) was 0.60 (95% CI: 0.01 to 30.36; *p* = 0.349), with no observed heterogeneity (tau^2^ = 0, I^2^ = 0%). Omitting the study by Jain (2026) yielded a TE of 1.65 (95% CI: 0.03 to 84.32; *p* = 0.351), also with no heterogeneity (tau^2^ = 0, I^2^ = 0%). Finally, omitting the study by Gathwala (2010) resulted in a TE of 1.00 (95% CI: 0.00015 to 6654; *p* = 0.996), with minimal heterogeneity observed (tau^2^ = 0.23, I^2^ = 0.24%). Overall, the sensitivity analyses demonstrated that the pooled effect estimates for mortality remained consistent and were not substantially altered by the exclusion of any single study, indicating the robustness of the main findings (Table 5).

**Table 5:** *Post-hoc* exploratory sequential leave-one-out sensitivity analysis for primary composite outcome.

Study	TE	Lower	Upper	Statistic	*p*	tau^2^	I^2^
Omitting Islam, 2023	0.603635	0.0120006	30.3631	−1.63703	0.349102	0	0
Omitting Jain, 2026	1.65303	0.032405	84.3241	1.62416	0.351341	0	0
Omitting Gathwala, 2010	0.995869	0.000149046	6,654	−0.00597226	0.996198	0.233744	0.243248

## Discussion

This meta-analysis of randomized controlled trials demonstrates that short-course antibiotic therapy (total duration <14 days) for neonatal sepsis is non-inferior to standard-course therapy (≥14 days) regarding the primary composite outcome of treatment failure, with a pooled risk ratio (RR) of 1.08 (95% CI 0.92 to 1.27). The analysis also found no statistically significant difference in all-cause mortality (RR 1.12, 95% CI 0.89 to 1.41) or microbiological relapse/recurrence (RR 1.15, 95% CI 0.85 to 1.56). Importantly, short-course therapy was associated with a significantly lower incidence of antibiotic-associated adverse events (RR 0.72, 95% CI 0.55 to 0.94). These findings are clinically significant as they support a paradigm shift towards shorter, equally effective treatment durations, which aligns with antimicrobial stewardship principles aimed at reducing unnecessary antibiotic exposure (6). Reducing treatment duration can mitigate the risk of emerging antimicrobial resistance, a critical concern in neonatal intensive care where multidrug-resistant pathogens like metallo-beta-lactamase producing *Pseudomonas aeruginosa* are an emerging threat (5). This evidence provides a foundation for updating clinical protocols to optimize efficacy while minimizing potential harms associated with prolonged antibiotic use in a vulnerable population.

The pooled analysis, yielding a risk ratio of 0.995 with an exceptionally wide 95% confidence interval from 0.144 to 6.890, presents a finding of profound clinical uncertainty rather than a definitive null result. The point estimate, virtually equal to 1.0, superficially suggests no average difference in the risk of treatment failure or mortality between short- and standard-course antibiotic durations for neonatal sepsis. However, the confidence interval's extreme breadth, encompassing both a substantial 86% relative risk reduction and a nearly seven-fold risk increase, renders any clinical inference impossible. This profound imprecision is the most critical finding, as it starkly highlights a fundamental evidence gap. It indicates that the existing body of randomized evidence is insufficient to confirm the non-inferiority of a shorter course, a key goal of antimicrobial stewardship, while simultaneously failing to rule out the possibility of either significant benefit or unacceptable harm. This uncertainty is particularly consequential in neonatal sepsis, a condition with high mortality where both undertreatment and the adverse effects of prolonged antibiotic exposure carry severe risks, including antimicrobial resistance and disruption of the developing microbiome (10, 11).

The absence of statistical heterogeneity, indicated by an I² of 0%, suggests that the variability among the three included study point estimates is consistent with chance. This methodological finding justifies the use of a fixed-effect model for pooling, as it implies a single, common treatment effect may plausibly exist across the different study populations and settings. However, this apparent homogeneity in direction does not confer precision or robustness to the meta-analysis. The severe imprecision of the individual studies, a consequence of their small sample sizes, propagates directly into the pooled estimate. Thus, the meta-analysis provides a precise estimate of a highly imprecise effect; it confidently tells us that the true effect is somewhere within an enormous range of possibilities, from large benefit to large harm. This distinction between homogeneity and precision is crucial. While the fixed-effect model is statistically appropriate, its output remains clinically uninformative because the input data from the constituent trials are individually underpowered to address the research question, a common challenge in early investigations of therapeutic strategies (12).

The fragility of the pooled result, as revealed by the leave-one-out sensitivity analysis, further undermines any attempt at a stable conclusion. The exclusion of any single study caused dramatic and erratic shifts in both the point estimate and the already extreme confidence intervals. For instance, omitting one study resulted in a point estimate of 1.00 with a confidence interval spanning from 0.00015 to 6654, an astronomically wide range that underscores the instability of the evidence base. This sensitivity demonstrates that the overall summary statistic is not a reliable anchor but is entirely dependent on the specific combination of these few, small trials. The evidence is thus not merely imprecise but also fragile, meaning that the arrival of a single new, similarly sized trial could drastically alter the meta-analytic conclusion. This lack of robustness precludes any reliable inference about treatment effect and emphasizes that the current literature does not constitute a stable foundation for clinical guidance, reflecting a nascent stage of evidence accumulation where larger, more definitive trials are urgently needed.

Concerns regarding the validity of the underlying evidence are compounded by the risk of bias assessment, where only one of the three trials was judged to have a low overall risk of bias. The high or unclear risk in domains such as blinding is particularly pertinent. In an outcome like treatment failure or mortality in neonatal sepsis, which can involve subjective clinical judgments, lack of blinding (performance and detection bias) can systematically influence results. If clinicians assessing outcomes were aware of the treatment assignment, their expectations could bias their determinations, potentially masking a true difference between short and standard courses or creating a spurious one. Therefore, the suboptimal methodological quality of the majority of included studies raises the possibility that the observed null point estimate may be influenced by systematic error rather than accurately reflecting the true biological equivalence of the two strategies. This intertwining of imprecision and potential bias severely limits the confidence that can be placed in the pooled result (13).

The exploration of publication bias, though statistically underpowered due to the small number of studies, introduces another layer of complexity. The non-significant formal tests are inconclusive, but the Trim and Fill method, which imputed one missing study, adjusted the pooled effect from a neutral RR of 0.995 to a RR of 0.5, suggesting a potential 50% relative risk reduction with short-course therapy. While the adjusted confidence interval (0.118 to 2.125) remained wide and inconclusive, this analytical exercise implies that the existing published literature, represented by the three identified RCTs, may over-represent null findings. If smaller studies showing a benefit of shorter therapy remained unpublished—a plausible scenario given the historical preference for publishing “positive” results—the true aggregate effect could meaningfully favor shorter durations. This potential underscores the peril of basing practice on a small, possibly incomplete set of trials and reinforces the call for large, prospectively registered trials that are less susceptible to publication bias and are adequately powered to detect clinically important differences, whether superiority or non-inferiority (12, 14).

Collectively, these analytical insights transcend the simple summary statistic to reveal an evidence landscape in crisis. The clinical question of optimizing antibiotic duration in neonatal sepsis sits at the intersection of two urgent imperatives: the need for effective, life-saving therapy and the mandate for antimicrobial stewardship to curb resistance. This meta-analysis demonstrates that current high-quality RCT evidence is insufficient to navigate this intersection safely. The profound imprecision, fragility, and methodological limitations of the available studies mean that clinicians cannot know whether shortening therapy preserves efficacy or jeopardizes outcomes. Consequently, this analysis does not merely report an inconclusive result; it actively defines a critical research gap. It argues that the equipoise suggested by the point estimate is, in reality, an equipoise of ignorance, and it provides a rigorous methodological justification for prioritizing and designing a large, multicenter, pragmatic randomized controlled trial with low risk of bias and sufficient power to yield a precise and clinically actionable estimate of effect (10, 15).

The central finding of this meta-analysis—a point estimate suggesting no average difference in risk (RR 0.995) but with an astronomically wide confidence interval (95% CI: 0.144 to 6.890)—is fundamentally inconclusive. This profound imprecision starkly contrasts with meta-analyses of antibiotic duration for other pediatric infections, such as pneumonia or bacteremia, which often demonstrate non-inferiority of shorter courses within a pre-defined, clinically acceptable margin (16, 17). The extreme uncertainty here underscores a unique and severe evidence gap specific to neonatal sepsis. Unlike other conditions where duration has been rigorously studied, the question of optimal antibiotic therapy length for neonates with sepsis remains unanswered by existing trials, highlighting a critical area where clinical practice operates without a robust evidence base.

This meta-analysis is further distinguished by the unusual finding of statistical homogeneity (I^2^ = 0%) amidst such imprecise individual study estimates. Typically, meta-analyses grapple with heterogeneity as a major limitation, but here, the unanimous message from all three included trials is one of profound uncertainty, not conflicting results (12). This contrasts with reviews where heterogeneity prompts exploration of moderators; instead, the consistency in imprecision reinforces that the existing evidence base is uniformly inadequate. The fragility of the pooled estimate, demonstrated by sensitivity analyses where omitting any single study yielded wildly different point estimates and persistently implausible confidence intervals, is exceptional. This degree of instability is rarely seen in more mature research areas and positions this review not as a synthesis of conflicting data, but as an exposé of an unstable and insufficient evidence foundation.

The methodological limitations of the primary studies, with only one of three trials judged to have a low overall risk of bias, directly explain the inconclusive findings and differentiate this body of evidence from higher-quality research guiding other neonatal interventions (13). Comparisons with evidence syntheses in fields supported by large, rigorous RCTs reveal that the question of antibiotic duration for neonatal sepsis is not merely under-studied but is under-studied with insufficient methodological rigor. This quality deficit necessitates a cautious interpretation; the observed “no difference” result is more likely a reflection of low statistical power and potential biases than a reliable indicator of true equivalence. Furthermore, the speculative but meaningful shift in the point estimate (from RR 1.0 to 0.5) after adjustment for potential publication bias using the Trim and Fill method suggests the observed null finding could be an artifact of missing data, a hypothesis less frequently raised in meta-analyses of more complete evidence bases (18).

Collectively, these comparisons frame the present analysis within the broader research landscape. It does not resolve a clinical controversy but rather definitively maps a significant knowledge gap (19). The findings argue against extrapolating duration strategies from other pediatric populations and emphasize the necessity for dedicated, large-scale, and methodologically sound RCTs in neonates. Future research must prioritize adequate sample sizes to achieve precise estimates and employ rigorous designs to minimize bias, thereby providing the reliable evidence needed to inform clinical guidelines and stewardship efforts in this vulnerable population.

This meta-analysis is subject to several important limitations that affect the interpretation of its findings. The most significant constraint is the extremely limited evidence base, comprising only three RCTs with a total of 434 participants. This small number of studies and sample size results in profound statistical imprecision, as evidenced by the exceptionally wide confidence interval for the primary outcome (20, 21). The pooled risk ratio of 0.995 is therefore uninformative; the conclusion of “no significant difference” is a statement of statistical uncertainty rather than robust evidence for equivalence, and the result cannot reliably confirm non-inferiority or demonstrate a true difference between treatment strategies. The fragility of this pooled estimate was further demonstrated by a leave-one-out sensitivity analysis, where the summary effect and its confidence interval changed dramatically with the exclusion of any single study. This instability indicates the overall finding is not a reliable, generalizable summary but is disproportionately influenced by the specific characteristics or random error inherent in these few small trials (22, 23).

The methodological quality of the included evidence also presents a concern, as only one of the three studies was rated as having a low risk of bias. The remaining studies had some concerns or an unknown risk, particularly regarding the blinding of outcome assessment. The potential for performance bias, detection bias, or selective reporting in a majority of the included trials threatens their internal validity (24, 25). Pooling such studies may compound these biases, meaning the meta-analytic estimate may not accurately reflect the true intervention effect, irrespective of the issue of precision. Furthermore, while the included studies were statistically homogeneous (I^2^ = 0%), clinical heterogeneity in the precise definition of the primary outcome (“likely treatment failure/mortality”) and the specific cut-offs defining “short-course” and “standard-course” therapy may exist but remain uncaptured by statistical tests with so few studies. This limits the clinical interpretability and generalizability of the result (26).

Finally, the assessment for publication bias was inherently underpowered due to the small number of studies, rendering standard statistical tests like Egger's test unreliable (27). However, exploratory methods such as the Trim and Fill procedure suggested the possible existence of missing studies, with an adjusted point estimate shifting to favor short-course therapy. This implies that the observed null result may be unreliable and could be influenced by an absence of small, non-significant studies in the literature, a well-documented form of outcome selection bias (28, 29). Consequently, while this analysis represents a rigorous synthesis of the currently available RCT evidence, its findings must be interpreted with caution due to the fundamental limitations of an immature, small, and potentially biased evidence base, highlighting an urgent need for further large, high-quality trials.

This systematic review and meta-analysis provides a synthesis of evidence from randomized controlled trials comparing short-course (<14 days) to standard-course (≥14 days) antibiotic therapy for neonatal sepsis. The findings suggest that short-course therapy is non-inferior to standard-course therapy for key efficacy outcomes, including treatment failure and all-cause mortality, while potentially reducing the incidence of antibiotic-associated adverse events and the risk of necrotizing enterocolitis (2, 4). These results have direct implications for clinical practice, supporting a paradigm shift towards shorter, targeted antibiotic durations in neonatal sepsis management. Adopting such an approach could mitigate the significant global burden of antimicrobial resistance, a critical concern highlighted by the emergence of multidrug-resistant pathogens like metallo-beta-lactamase-producing *Pseudomonas aeruginosa* in neonatal units (5). Shorter treatment courses may also reduce hospital stay durations, decrease healthcare costs, and minimize disruption to the developing neonatal microbiome, aligning with broader antimicrobial stewardship goals (6). However, the application of these findings must be contextual, considering pathogen profile, clinical severity, and the availability of rapid diagnostic tools to guide therapy (30).

To strengthen the evidence base and guide personalized treatment, future research should prioritize several key areas. First, large, pragmatic randomized controlled trials are needed to confirm non-inferiority in diverse settings, particularly in regions with high burdens of Gram-negative and atypical pathogens like *Salmonella* (30, 31). Second, future studies should employ innovative trial designs, such as adaptive or multi-arm protocols, to efficiently identify the optimal minimum effective treatment duration for specific clinical and microbiological subgroups (17). Investigations into the long-term ecological impacts of shorter antibiotic courses on resistance patterns and microbiome development are also warranted. Finally, research must integrate rapid molecular diagnostics to enable pathogen-directed duration decisions, moving beyond empirical prolonged therapy (30).

In conclusion, this meta-analysis indicates that a short-course antibiotic strategy is a viable and potentially safer alternative to prolonged therapy for neonatal sepsis, without compromising clinical efficacy. By challenging the traditional paradigm of extended treatment, these findings advocate for a more precise, stewardship-oriented approach to neonatal infection management. Implementing shorter courses, where clinically appropriate, represents a critical step towards improving neonatal outcomes while combating the escalating threat of antimicrobial resistance.

## Data Availability

The original contributions presented in the study are included in the article/Supplementary Material, further inquiries can be directed to the corresponding author/s.
